# A decade of artificial intelligence research in ophthalmology: Global trends and transferable insights for medical AI

**DOI:** 10.1371/journal.pdig.0001347

**Published:** 2026-08-25

**Authors:** Zeyue Fan, Yiming Qin, Luxiao Chen, Jixuan Yuan, Zhuxin Xiong, Xiaodong Chen, Shixin Lai, Yih Chung Tham, Bin Sheng, Xiaofei Wang, Andrzej Grzybowski, Aaron Y. Lee, Cecilia S. Lee, Carol Y. Cheung, T. Y. Alvin Liu, Tien Yin Wong, Ya Xing Wang

**Affiliations:** 1 Beijing Visual Science and Translational Eye Research Institute (BERI), Beijing Tsinghua Changgung Hospital Eye Center, Tsinghua Medicine, Tsinghua University, Beijing, China; 2 Beijing Key Laboratory of Intelligent Diagnostic Technology and Devices for Major Blinding Eye Diseases, Tsinghua Medicine, Tsinghua University, Beijing, China; 3 Key Laboratory for Biomechanics and Mechanobiology of Ministry of Education, Beijing Advanced Innovation Center for Biomedical Engineering, School of Biological Science and Medical Engineering, Beihang University, Beijing, China; 4 Singapore Eye Research Institute, Singapore National Eye Center, Singapore, Singapore; 5 Centre for Innovation and Precision Eye Health, Department of Ophthalmology, Yong Loo Lin School of Medicine, National University of Singapore, Singapore, Singapore; 6 Ophthalmology and Visual Sciences Academic Clinical Program, Duke-NUS Medical School, Singapore, Singapore; 7 School of Computer Science, Shanghai Jiao Tong University, Shanghai, China; 8 MoE Key Lab of Artificial Intelligence, AI Institute, Shanghai Jiao Tong University, Shanghai, China; 9 Department of Ophthalmology, University of Warmia and Mazury, Olsztyn, Poland; 10 Institute for Research in Ophthalmology, Foundation for Ophthalmology Development, Poznan, Poland; 11 John F. Hardesty MD Department of Ophthalmology and Visual Sciences, Washington University in St. Louis, St. Louis, Missouri, United States of America; 12 Department of Ophthalmology and Visual Sciences, The Chinese University of Hong Kong, Hong Kong, China; 13 Wilmer Eye Institute, School of Medicine, Johns Hopkins University, Baltimore, Maryland, United States of America; London School of Hygiene & Tropical Medicine, UNITED KINGDOM OF GREAT BRITAIN AND NORTHERN IRELAND

## Abstract

To characterize global trends in ophthalmic AI research from 2015–2025 and drive transferable insights into the broader evolution of AI in medicine, we conducted a systematic bibliometric analysis of original AI articles in ophthalmology indexed in the Web of Science Core Collection, Scopus, and Pubmed from 2015 to 2025. Publications were screened and categorized using an LLM-assisted pipeline with predefined labels, and agreement between LLM-assisted classifications and human grading was evaluated. We analyzed temporal trends and emergence patterns across study design, model architecture, disease focus, and data modalities. Among 12,911 included articles, annual publications increased at a compound annual growth rate of 39.7%, from 108 in 2015–3061 in 2025. Four key shifts were identified. Study design: Development studies (88.3%) dominated through the period, whereas evaluation studies (7.4%) began to emerge in 2019. Model architecture: Classical deep learning (71.8%) became the most prevailing approach from 2017, while foundation model studies (0.6%) increased sharply in 2024–2025. Disease focus: Diabetic retinopathy (32.9%), glaucoma (17.5%), and age-related macular degeneration (12.6%) remained the leading disease areas, while corneal diseases (7.0%), cataract (4.3%) and myopia (3.4%) emerged after 2019–2020 and grew rapidly. Data modalities: Among image-based modalities (84.1%), color fundus photography and retinal optical coherence tomography remained dominant but plateaued after 2019, whereas text-based modalities (5.5%) continued to rise after 2022. Over the past decade, ophthalmic AI research expanded rapidly and evolved from narrow image-based deep learning toward broader work spanning evaluation studies, foundation models, multimodal data integration, and a wider spectrum of eye diseases. These trajectories may extend beyond ophthalmology, offering broader insights into how medical AI matures toward clinical evaluation and translation.

## Author summary

Artificial intelligence (AI) research has expanded rapidly in ophthalmology over the past decade, supported by the field’s rich imaging data and the availability of several well-defined clinical tasks. Yet it remains unclear how ophthalmic AI has evolved over time, and what this trajectory can teach the wider medical AI community. In this study, we systematically analyzed 12,911 original research articles published between 2015 and 2025 to map the global development of AI in ophthalmology. We found that over time, the field broadened in several important ways: evaluation studies became more common; foundation models and multimodal approaches began to emerge; text-based inputs increased; and research expanded to a wider range of eye diseases such as corneal disease, cataract, and myopia. These findings suggest that ophthalmic AI is moving from a narrow focus on image-based detection toward more generalizable, clinically relevant, and workflow-oriented applications. More broadly, this evolution may provide a useful example of how medical AI matures from technical feasibility, to broader disease coverage and multimodal integration, and ultimately toward real-world clinical translation.

## Introduction

Ophthalmology has become one of the most active clinical specialties in the development and potential adoption and integration of artificial intelligence (AI) in medicine. Across medical subfields, it ranks among the leaders in both the volume and impact of AI-related research [1,2].

This momentum is enabled by the field’s image-rich workflows (e.g., color fundus photographs [CFP]) and by clinically meaningful, well-defined diagnostic endpoints such as referable diabetic retinopathy (DR) [3,4]. Over the past decade, ophthalmology was also among the first specialties to demonstrate the clinical potential of deep learning at scale, particularly for DR screening [5–11]. Notably, IDx DR became the first FDA-approved autonomous AI model for medical diagnosis [12]. In parallel, implementation activity has accelerated. A recent scoping review identified 36 regulatory-authority approved ophthalmic AI software as medical device products across the United States, European Union (EU), and Australia [13].

These advances position ophthalmology not only as an early adopter of clinical AI, but also as a practical model for both image-intensive and non-image-intensive specialties seeking to translate algorithmic innovation into real-world value. Meanwhile, medical AI has rapidly evolved, with the introduction of large language models (LLMs) such as ChatGPT and transformer-based foundation models reshaping how models are trained, evaluated, and deployed. As a result, ophthalmic AI research has progressed from early machine-learning applications to deep learning and, more recently, to multimodal approaches and foundation-model. Mapping this evolution provides an opportunity to derive transferable lessons that may help accelerate responsible AI development, evaluation, and adoption across medicine and digital health [14,15].

To address this need, we conducted a systematic bibliometric analysis of ophthalmic AI research from 2015 to 2025, focusing on four key dimensions: (1) the types of development and evaluation studies conducted; (2) the architectures of the models developed; (3) the research focus across different ophthalmic diseases; and (4) the distribution of input modalities across different study types. As a supporting method to enable large-scale screening and classification of eligible studies, we implemented an LLM-assisted review pipeline using gpt-5-mini model from OpenAI and evaluated agreement against human grading. Our findings offer a data-driven map of the field’s evolution and an empirical basis for benchmarking medical AI research trajectories.

## Results

**Fig 1:**
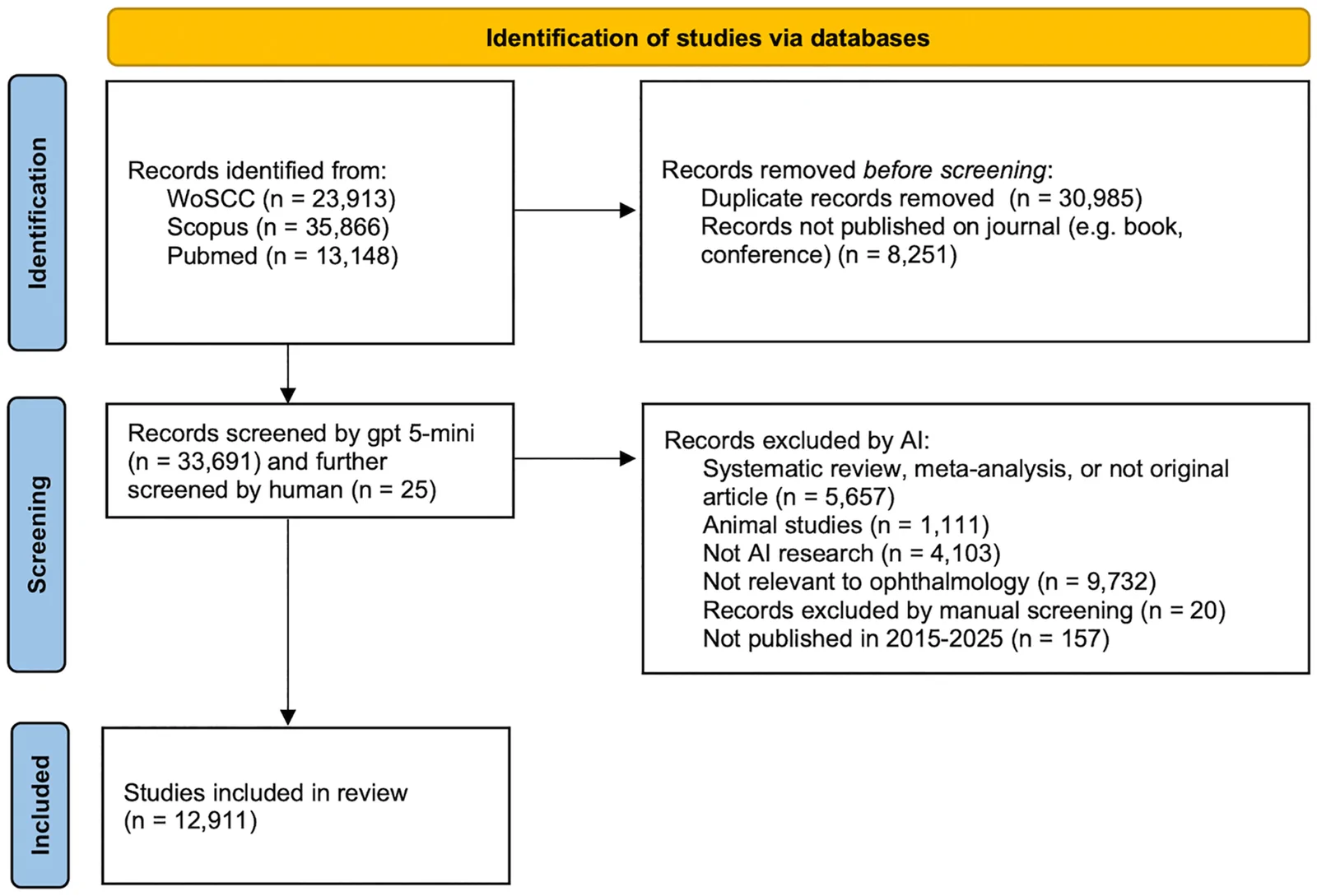
The data collection and retrieval strategy.

**Fig 2:**
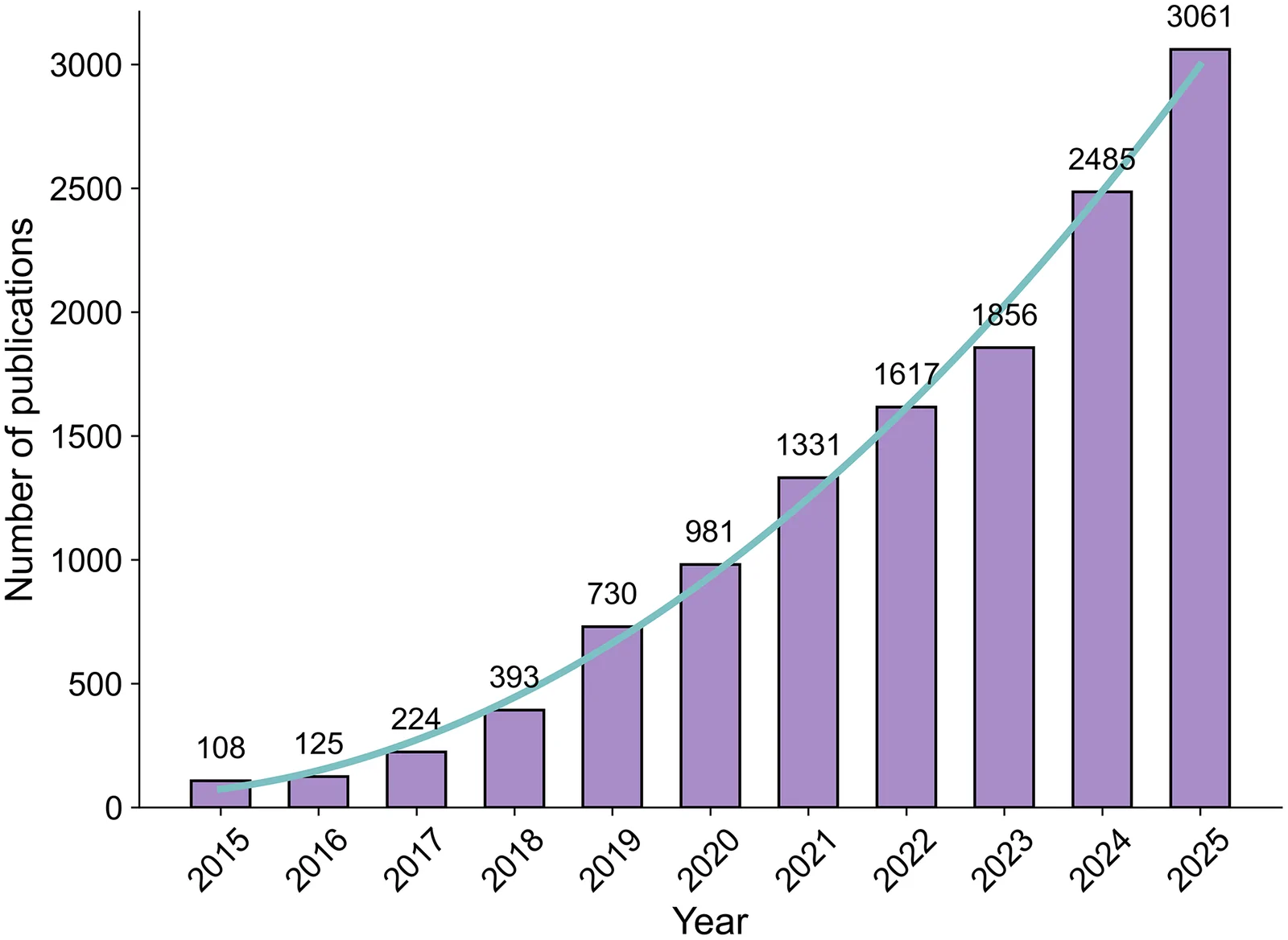
Annual number of publications of AI in ophthalmology from 2015 to 2025.

Between 2015 and 2025, a total of 33,691 articles in ophthalmic AI were initially identified, and 12,911 met the inclusion criteria for analysis (Fig 1). In the inclusion/exclusion task, the agreement between the LLM-assisted pipeline and human annotations was high (Cohen’s κ = 0.92). The number of publications increased from 108 in 2015–3,061 in 2025, corresponding to a compound annual growth rate of 39.7% (Fig 2).

### 1. Trends in development vs. evaluation studies

**Fig 3:**
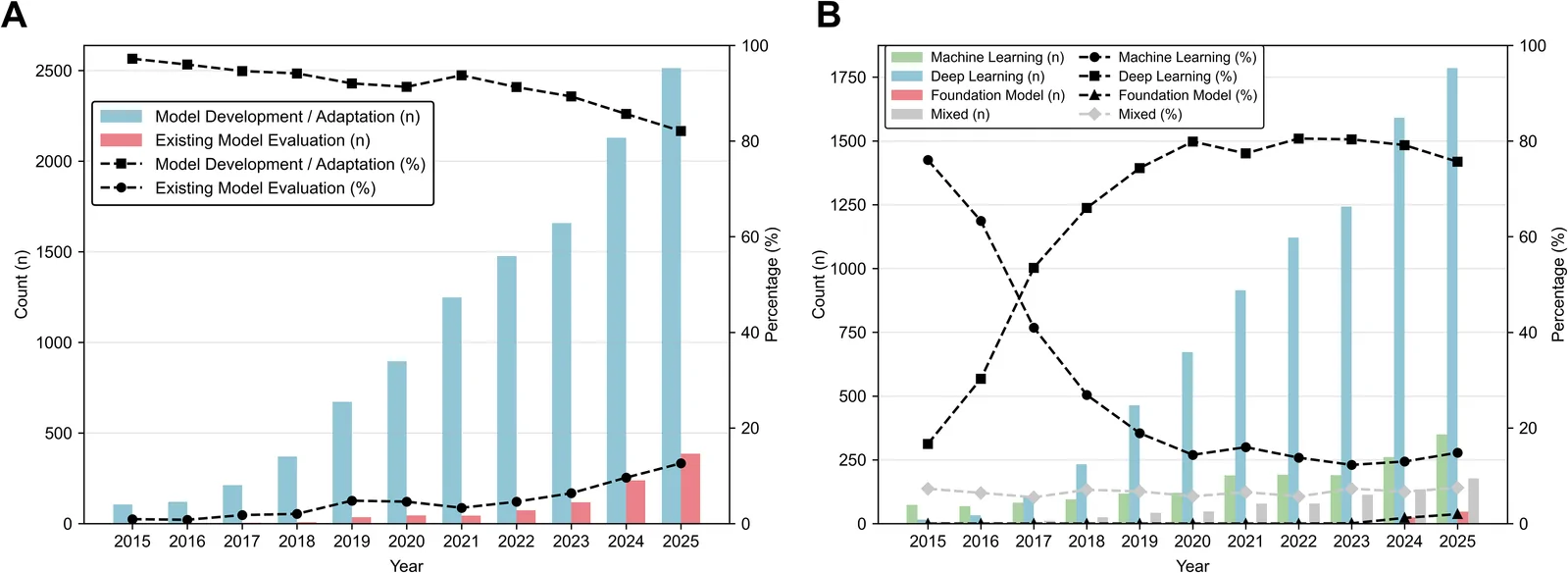
Trends and comparative analysis of study design and model architecture of ophthalmic AI research. **(A)** Annual number and proportion of publications categorized by study design: model development vs. evaluation; **(B)** Annual number and proportion of AI model architectures used in development studies.

Of the included studies, 11,399 (88.3%) focused on developing AI models, while 955 (7.4%) evaluated existing models. The proportion of valuation studies increased in two waves, with an initial rise in 2019 followed by a more sustained upward trend after 2021. The proportion of development studies generally declined over time, despite a transient increase in 2021 (Fig 3A).

### 2. Trends in model types

Among 11,399 development studies, 1,740 studies (15.3%) used machine learning, 8,180 (71.8%) used deep learning, and 72 (0.6%) adopted foundation models. Machine learning emerged earliest, but deep learning (30.3% in 2016 and 53.5% in 2017) rapidly surpassed machine learning (63.3% in 2016 and 41.0% in 2017) and has remained the dominant approach since 2017. Meanwhile, the proportion of machine learning studies continued to decrease before plateauing after 2020 (12.3%-16.0%). The share of deep learning studies also declined modestly (from 80.5% to 75.7%) after 2022. Foundation models first appeared in ophthalmology in 2023, expanded to more than 20 studies in 2024, and nearly doubled in 2025 (Fig 3B).

### 3. Trends in disease focus

**Fig 4:**
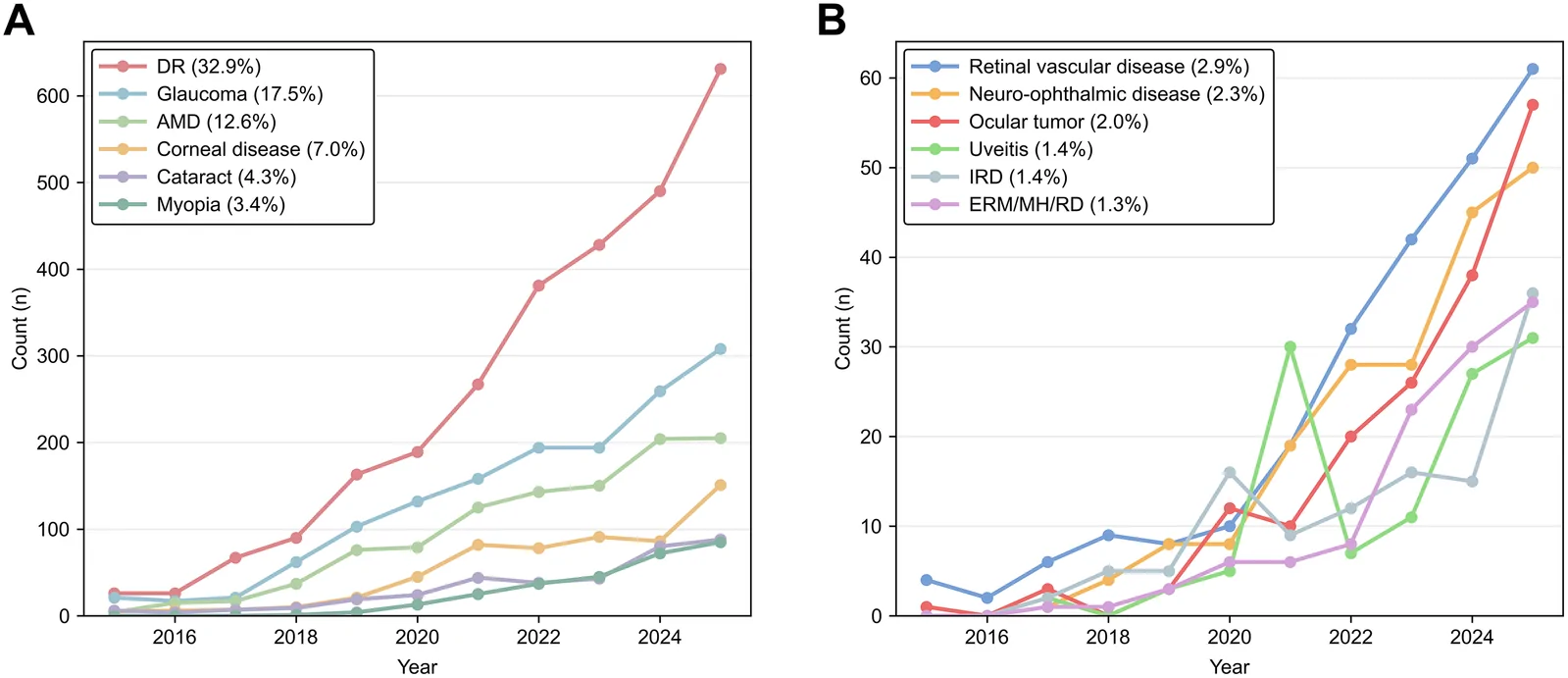
Trends and comparative analysis of disease-specific research focus in ophthalmic AI research. **(A)-(B)** Distribution of diseases among disease-specific studies and annual number of publications categorized by disease among disease-specific studies. DR, diabetic retinopathy; AMD, age-related macular degeneration; IRD, inherited retinal disease; ERM, epiretinal membrane; MH, macular hole; RD, retinal detachment.

Among all included studies, 64.9% focused on a specific disease, whereas 12.0% were addressing broader disease categories (e.g., retinal diseases) or general ophthalmic or multiple diseases that could not be classified into a single subspecialty, and 23.2% on non-disease-specific topics (e.g., image quality, segmentation, methodological studies). Among the 8,374 studies focused on a specific disease, the top six categories were diabetic retinal diseases (32.9%), glaucoma and optic nerve head diseases (17.5%), macular and retinal degeneration (12.6%), corneal and ocular surface diseases (7.0%), cataract and lens disorders (4.3%), and myopia and pathological myopia (3.4%). The remaining six categories, although less prevalent, also exhibited substantial growth, including retinal vascular diseases (2.9%), neuro-ophthalmic diseases and non-glaucomatous optic neuropathies (2.3%), ocular tumor and oncology (2.0%), followed by inherited retinal diseases (1.4%), uveitis and intraocular inflammatory diseases (1.4%), and vitreoretinal interface diseases and retinal detachment (1.3%) (Fig 4A-B). The temporal trends revealed a marked increase in DR starting in 2017, followed by glaucoma and age-related macular degeneration (AMD), which showed clear growth beginning in 2018. The order of emergence of other disease categories can also be readily observed from the temporal patterns (Fig 4A-B).

### 4. Trends in modalities

**Fig 5:**
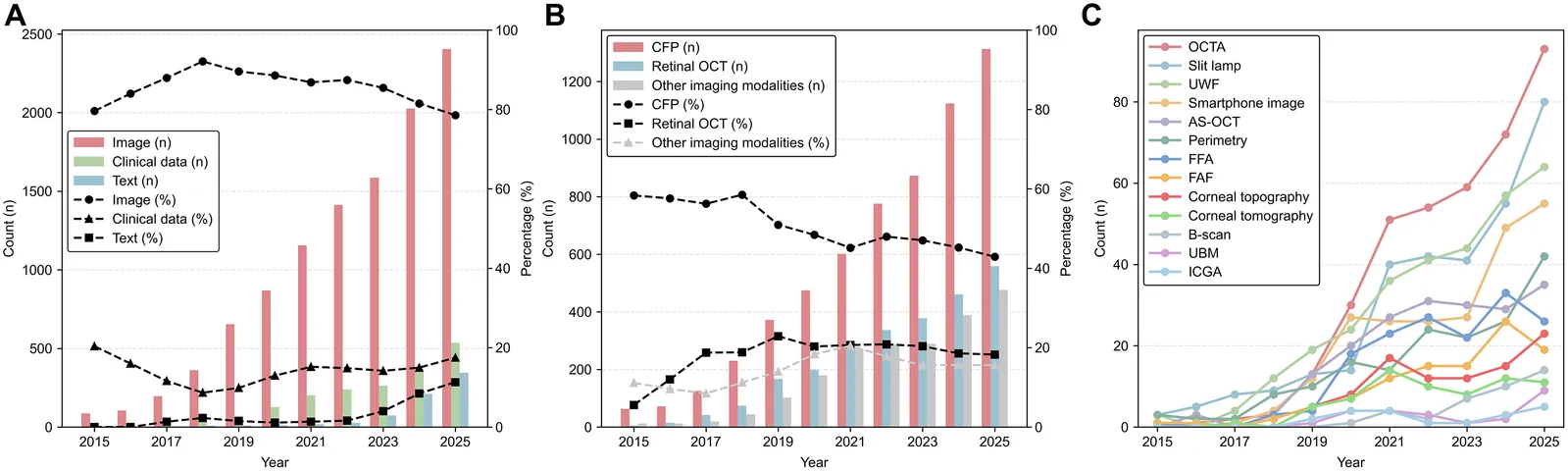
Trends and comparative analysis of input modalities in ophthalmic AI research. **(A)** Annual number and proportion of publications categorized by input data modalities; **(B)** Annual number and proportion of publications categorized by detailed image input modalities (CFP, OCT and others); **(C)** Annual number of publications categorized by detailed image input modalities.

Overall, 84.1% of studies involved image modalities, compared with 14.8% using clinical data and 5.5% using text. Temporal analysis showed that the use of text increased notably from 2023 onward. The proportion of studies incorporating clinical data declined between 2015 and 2018, whereas image-based modalities increased substantially over the same period. From 2018 onward, these trends became attenuated and gradually shifted in opposite directions, with a slight increase in the use of clinical data and a slight decrease in image-based modalities (Fig 5A).

When categorized according to single-modality and multi-modality approaches, single-modality studies accounted for the majority (91.1%), including single-input single-modality (72.8%) and multi-view/multi-slice/longitudinal single-modality (18.3%). Multi-modality studies comprised a smaller proportion (8.9%), including cross-domain multi-modality (5.0%) and within-imaging multi-modality (3.8%). Among image-based modalities, CFP (55.5%) and retinal optical coherence tomography (OCT) (23.1%) were the predominant modalities, each individually surpassing the combined total of all other modalities in almost every year. CFP has the longest history of use, whereas OCT showed a notable increase from 2015 to 2019, after which the use of both modalities remained largely stable (CFP ~ 45%; retinal OCT ~ 20%) (Fig 5B). Optical coherence tomography angiography (OCTA), slit-lamp photography, ultra-widefield (UWF) imaging, and smartphone-based external eye photography were the other four modalities with relatively high proportions (Fig 5C).

## Discussion

With a systematic bibliometric analysis of ophthalmic AI research from 2015 to 2025, we quantified growth and mapped temporal changes in study design, model architecture, disease focus, and data modalities. Among 12,911 included original articles, annual output increased from 108 in 2015–3061 in 2025 (39.7% compound annual growth), underscoring the rapid expansion of the field. Scientifically, ophthalmic AI remained dominated by development studies and classical deep learning, with sustained emphasis on diabetic retinopathy, glaucoma, and AMD, and predominant use of CFP and OCT. However, the later years of the decade showed clear shifts, including an increase in evaluation studies, the emergence and rapid rise of foundation-model work in 2024–2025, growth in text-based approaches, and expanding attention to some previously-less-studied but common diseases, like corneal diseases, cataract and myopia.

These transitions suggest three broader, hypothesis-generating lessons. We acknowledge that ophthalmology benefits from domain-specific advantages, including abundant imaging data, relatively standardized annotation targets, and established screening use cases. These conditions may not extend uniformly to fields with different data types, clinical workflows, and annotation challenges, such as psychiatry or primary care. Accordingly, the following lessons should be interpreted not as universally generalizable claims, but as patterns emerging from ophthalmology that may help inform broader thinking about how medical AI moves from technical development toward clinical evaluation and implementation.

First, disease selection in medical AI often progresses from data-rich, well-defined targets to conditions with greater diagnostic complexity or weaker labeling infrastructure. For leading blindness-causing eye diseases, early work concentrated on DR [5–11,16–20], AMD [21,22], and glaucoma [23], but attention has expanded to cataract [24–26] and pathologic myopia [27]. Additionally, by leveraging imaging and AI, oculomics extends ophthalmology’s translational paradigm from organ-specific screening to pan-disease risk stratification, and may deepen the integration of AI into systemic disease management in yet broader care pathways [28–30].

Second, dominant data types shape a field’s AI trajectory. The long-standing primacy of CFP and OCT has anchored ophthalmic AI in screening-oriented applications, reflected by the sustained dominance of image-based development studies throughout the decade [5–8,11,18–23,31]. The recent growth of text-based approaches and multi-modal inputs may signal a shift toward decision support for more complex clinical reasoning, where models integrate imaging with clinical history, demographics, symptoms, and narrative documentation [32–39]. If sustained, this transition could expand ophthalmic AI from single-task detection toward more comprehensive assistance across referral decisions, patient communication, and follow-up planning.

Third, foundation models may enable a step change from task-specific systems to more general-purpose ophthalmic AI. We observed a sharp increase in foundation-model studies in 2024–2025, consistent with the emerging use of both LLMs and vision-language models in ophthalmology [40–42]. RETFound represents a landmark retinal foundation model and, importantly, was released openly, facilitating downstream adaptation, benchmarking, and comparison across tasks and datasets [43,44]. Parallel efforts of multi-modality foundation models [36,37,39,45] suggest movement toward broader disease coverage and a wider range of imaging inputs. Building on these developments, large multi-modal models that combine diverse imaging with text and structured clinical variables may better reflect real-world clinical workflows.

Ultimately, the field’s growth will be judged by its ability to bridge the “last-mile” gap between publication and routine clinical use. Ophthalmology has made tangible progress in DR screening, where multiple autonomous systems have obtained FDA and EU clearance [12,46]. Yet regulatory authorization has not translated into widespread adoption, and the scale of model development and evaluation remains mismatched with real-world implementation [47]. Bridging this gap requires coordinated changes: earlier clinician involvement, prospective workflow-embedded evidence generation, and healthcare-specific standards for safety, equity, usability, and integration [48,49]. Evaluation should extend beyond benchmarks and follow a graded evidence pathway, progressing from retrospective validation to prospective observational studies, randomized or quasi-experimental outcome trials, and implementation research. In this context, the observed rise in evaluation-only studies may reflect maturation from feasibility demonstration toward assessing clinical readiness. Together, these patterns indicate a broadening research landscape within ophthalmic AI and provide a basis for considering which lessons may be relevant to medical AI more broadly (Table 1, Table 2).

**Table 1:** Developmental lessons (how medical AI evolves) from ophthalmic AI to other medical domains.

Domain	Evidence from Ophthalmic AI	Transferable Lesson
***Disease Targets***	Initial focus on DR, AMD, glaucoma → later cataract and pathologic myopia; expansion toward oculomics and systemic risk prediction	Medical AI typically progresses from data-rich, well-defined targets to diagnostically complex or weakly labeled conditions, and may eventually extend beyond organ-specific tasks
***Data Modalities***	Decade-long dominance of CFP/OCT image-based models; recent growth of multi-modal and text-integrated approaches	Dominant data types shape the trajectory and ceiling of AI applications, with multi-modality enabling transition from screening to clinical decision support
***Model Architectures***	Rapid rise of foundation models (e.g., RETFound) and multi-modal architectures	Foundation models drive a shift from task-specific systems to general-purpose clinical AI capable of broader workflows

**Table 2:** Translational lesson (how medical AI matures clinically) from ophthalmic AI to other medical domains.

Translational Stage	Evidence from Ophthalmic AI	Transferable Lesson
Evidence maturity (feasibility → readiness)	Growing proportion of evaluation-only and observational studies beyond model development studies	Medical AI fields mature by progressing from technical feasibility toward assessing real-world clinical readiness
Implementation and impact (readiness → benefit)	Regulatory approvals achieved but adoption limited; need for workflow-embedded and outcome-based trials	Clinical value depends on overcoming the last-mile gap through workflow integration and an evidence ladder culminating in outcome validation

This study provides a tool-agnostic bibliometric framework to map ophthalmic AI research over the past decade, enabling granular analyses across dimensions that are difficult to scale with manual review alone. While informed by traditional bibliometric approaches, our workflow extends conventional pipelines by leveraging LLMs for structured information extraction and classification, thereby improving scalability for longitudinal mapping. Nevertheless, this methodological innovation comes with inherent constraints that warrant careful consideration. First, we used the OpenAI API for automated article tagging rather than large-scale manual review. Although we performed additional human validation across all LLM-assisted classification tasks and observed high agreement, residual misclassification remains possible because validation was conducted on a random sample rather than through full manual relabeling of all included studies. In addition to these method-specific limitations, our analysis was restricted to indexed original articles and excluded grey literature, including unpublished studies and conference proceedings. This decision improved reproducibility and metadata consistency, but may have led us to overlook emerging trends that often appear first in conference venues or other non-indexed sources, particularly in rapidly evolving areas such as foundation models and multi-modal AI. Although this limitation is unlikely to fully explain the major long-term trends observed over the decade, the timing and magnitude of newer trends should be interpreted cautiously. Finally, caution is needed when extending these ophthalmology-derived patterns to other medical fields. Our study was not designed as a comparative cross-specialty bibliometric analysis. Therefore, the proposed lessons should be interpreted as hypothesis-generating insights rather than empirically confirmed principles generalizable to all medical fields.

In conclusion, this decade-long bibliometric analysis shows that ophthalmic AI research has expanded rapidly and undergone clear paradigm shifts, with LLMs and foundation models shifting the field from narrow image-based development toward broader evaluation and multi-modal integration. Yet, as in many areas of medical AI, publication growth does not automatically translate into routine clinical adoption. Looking ahead, progress will require rigorous external validation, greater emphasis on safety and usability, and stronger collaboration among AI researchers, clinicians, and health systems to translate innovation into reliable, patient-centered tools.

## Methods

### 1. Data source, searching strategies and inclusion/exclusion criteria

A systematic search was performed in the Web of Science Core Collection (WoSCC), Scopus and Pubmed to identify relevant publications related to AI in ophthalmology from 2015 to 2025. The complete searching strategy was showed in S1 Table. The data were retrieved between March 5 and March 6, 2026. We excluded non-article/non-original-investigation publication (e.g., reviews, editorials, letters), records not relevant to AI or ophthalmology, and animal studies (detailed inclusion/exclusion criteria showed in S1 Table). This screening yielded a dataset of original research articles directly addressing AI applications in ophthalmology.

### 2. Mult-criteria classification and temporal trend analysis

We classified the included articles using a predefined framework (S2 Table) across four dimensions: study design (e.g., model development or adaptation study, evaluation studies of existing pre-trained models), model architecture (e.g., conventional machine learning, deep learning, foundation model), disease focus, and input data modality.

**Study design.** Articles were categorized as model development or adaption if they involved any model training or fine-tuning, and as model evaluation if they tested an existing, off-the-shelf model without any training or fine-tuning. Because many development studies report internal and/or external validation, we labeled a study as “evaluation” only when the work focused solely on benchmarking a pre-existing model.

**Model architecture** (development studies only). Each development article was assigned to one mutually exclusive category: (1) classical machine learning (non–neural-network methods such as support vector machines, random forests, and logistic regression), (2) classical deep learning (neural networks that are not foundation models), or (3) foundation models. We operationally defined foundation models as large-scale pre-trained models that were designed or used as reusable backbones for adaptation. To be classified as a foundation model, a study had to meet the following criteria: the model was pre-trained on broad, large-scale, or heterogeneous data using task-agnostic, self-supervised, contrastive, generative, or multitask objectives; it was intended to support adaptation across multiple downstream tasks; and it was applied through prompting, zero-/few-shot inference, fine-tuning, or other adaptation strategies. Examples included retinal foundation models such as RETFound, ophthalmic generalist or vision-language models, and LLM-based ophthalmology systems. Throughout this paper, “machine learning” refers to classical machine learning (excluding deep learning), and “deep learning” refers to classical deep learning (excluding foundation models).

**Disease focus**. Disease focus was classified in two steps. First, each study was assigned to one of three study scope categories: specific disease, multiple/general ophthalmic diseases, or non-disease-specific. Second, studies classified as specific disease were further assigned to the single most appropriate disease category from a predefined list of 13 categories, including (1) diabetic retinal diseases, (2) macular and retinal degeneration, (3) glaucoma and optic nerve head diseases, (4) myopia and pathologic myopia, (5) cataract and lens disorder, (6) corneal and ocular surface diseases, (7) retinal vascular diseases, (8) vitreoretinal interface diseases and retinal detachment, (9) inherited retinal diseases, (10) neuro-ophthalmic diseases and non-glaucomatous optic neuropathies, (11) uveitis and intraocular inflammatory diseases, (12) ocular tumor and oncology, and (13) other specific disease.

**Data modality**. We identified the input data type as text, image, structured clinical data or others. For image-based studies, we further recorded whether the study used the following image modalities: (1) color fundus photography, (2) (retinal) optical coherence tomography, (3) anterior segment optical coherence tomography, (4) wide-field or ultra-widefield fundus imaging, (5) optical coherence tomography angiography, (6) slit-lamp photography, (7) standard automated perimetry, (8) corneal topography, (9) corneal tomography, (10) fluorescein angiography, (11) indocyanine green angiography, (12) fundus autofluorescence, (13) ultrasound biomicroscopy, (14) B-scan ocular ultrasonography, and (15) smartphone-based ophthalmic imaging.

**Temporal trends**. After classification, we quantified annual trends from 2015 to 2025 across study design, modal architecture, disease focus, and data modalities, and generated category-level visualizations. Annual numbers and proportions were measured. Proportions of study types and modalities were calculated relative to the total number of included publications in each year. Proportions of model architectures were calculated relative to the total number of model development studies in that year. Proportions of diseases were calculated relative to the total number of studies focusing on a specific disease. Figures were produced using Python (version 3.13) and R (version 4.5.2).

### 3. LLM-assisted screening and agreement with human annotation

We utilized OpenAI’s GPT-5-mini, which has been officially described as being suited for well-defined tasks and precise prompts, to facilitate efficient screening and classification of a large number of publications. A structured prompt was designed to assess inclusion eligibility and make classifications based on titles and abstracts. The full code and prompt used for this data extraction are available in our GitHub repository (link: https://github.com/fanzeyue-biblioAIiO/biblio-Ophthalmic-AI)*.* To evaluate the performance of automatic screening, we randomly sampled 200 papers and compared the LLM-assigned inclusion/exclusion tags against independent, blinded dual-reviewer annotations, with each paper annotated by two reviewers from a reviewer team (Z.F., L.C., J.Y., Z.X., X.C., and S.L.), and discrepancies were resolved through discussion. Agreement between human and AI was quantified using Cohen’s kappa. Papers that had not been included or excluded in automatic screening were independently annotated by two reviewers (Z.F. and S.L.) in a blinded manner. To validate the classification pipeline, we randomly selected 100 articles classified by the AI as “Include” for independent review by two reviewers from the same reviewer team. Manual review identified 2 articles that should have been excluded; the remaining 98 articles were subsequently annotated for all applicable downstream classification tasks. For all tasks with at least one disagreement, confusion matrices and Cohen’s κ statistics are reported in S1 Fig, with κ values ranging from 0.85 to 0.97, and all but two exceeding 0.90. To further assess performance for highly imbalanced categories, all positive cases were manually reviewed for the foundation model category and for categories with fewer than 50 AI-positive articles, whereas 50 AI-positive articles were randomly sampled for image modality categories with more than 50 positive cases. Across these targeted validations, the proportion of false-positive predictions among AI-positive cases was no greater than 4%. The resulting structured dataset was used for subsequent analyses.

## Supporting information

S1 Table Search terms and prompts. (DOCX)

S2 Table Labeling schema for LLM-assisted classification. (DOCX)

S1 Fig Confusion matrices and Cohen’s κ for human validation of AI-generated annotations across multiple classification tasks. (DOCX)
