# Supplementary material for: A decade of artificial intelligence research in ophthalmology: Global trends and transferable insights for medical AI
Source: PLOS Digit Health. 2026 Aug 25;5(8):e0001347. doi: 10.1371/journal.pdig.0001347 (PMC13505936; doi:10.1371/journal.pdig.0001347)
Supplement: Supplementary file 1 [file pdig.0001347.s001.docx]

**S1 Table. Search Terms and Prompts**

1. **Search terms**

**WoSCC**:

(TS=((artificial intelligence OR machine learning OR deep learning OR "large language model*" OR transformer* OR "foundation model*" OR "AI agent*" OR robot* OR "natural language process*" OR "supervised learning" OR "unsupervised learning" OR "self-supervised learning" OR "reinforcement learning"))) AND TS=((ocular* OR ophthal* OR orbital* OR intraocular* OR "eye disease*" OR "eye disorder*" OR "eye injur*" OR "eye problem*" OR fundus OR cornea* OR conjunctiv* OR choroid* OR sclera* OR retina* OR macula* OR vitreous OR vitreoretinal OR "optic disc*" OR "optic nerve*" OR eyelid* OR "extraocular muscle*" OR "visual pathway*" OR cataract* OR glaucoma* OR uveitis OR iritis OR kerato* OR keratitis OR blepharit* OR trachoma* OR retinopath* OR amblyopia OR strabismus OR anophthalmia OR anisocoria OR "optic neuritis" OR papilledema OR nystagmus OR myopia OR hyperopia OR astigmatism OR ametropia OR "refractive error*" OR "refractive disorder*" OR "axial length" OR orthokeratology OR atropine OR blindness OR "vision loss" OR "visual loss" OR "visual impair*" OR "vision defect*" OR "vision disorder*" OR "impaired vision" OR "optical coherence tomography" OR "fundus photograph*" OR "fluorescein angiograph*" OR "visual field*" OR perimetry OR vitrectom* OR phacoemulsification OR "intravitreal inject*" OR anti-VEGF)) AND PY=(2015-2025) AND LA=(English)

**Scopus**:

TITLE-ABS-KEY("artificial intelligence" OR "machine learning" OR "deep learning" OR "large language model*" OR transformer* OR "foundation model*" OR "AI agent*" OR robot* OR "natural language process*" OR "supervised learning" OR "unsupervised learning" OR "self-supervised learning" OR "reinforcement learning") AND TITLE-ABS-KEY(ocular* OR ophthal* OR orbital* OR intraocular* OR "eye disease*" OR "eye disorder*" OR "eye injur*" OR "eye problem*" OR fundus OR cornea* OR conjunctiv* OR choroid* OR sclera* OR retina* OR macula* OR vitreous OR vitreoretinal OR "optic disc*" OR "optic nerve*" OR eyelid* OR "extraocular muscle*" OR "visual pathway*" OR cataract* OR glaucoma* OR uveitis OR iritis OR kerato* OR keratitis OR blepharit* OR trachoma* OR retinopath* OR amblyopia OR strabismus OR anophthalmia OR anisocoria OR "optic neuritis" OR papilledema OR nystagmus OR myopia OR hyperopia OR astigmatism OR ametropia OR "refractive error*" OR "refractive disorder*" OR "axial length" OR orthokeratology OR atropine OR blindness OR "vision loss" OR "visual loss" OR "visual impair*" OR "vision defect*" OR "vision disorder*" OR "impaired vision" OR "optical coherence tomography" OR "fundus photograph*" OR "fluorescein angiograph*" OR "visual field*" OR perimetry OR vitrectom* OR phacoemulsification OR "intravitreal inject*" OR "anti-VEGF") AND PUBYEAR > 2014 AND PUBYEAR < 2026 AND ( LIMIT-TO ( LANGUAGE,"English" ) )

**Pubmed**:

((( ("Artificial Intelligence"[Mesh] OR "Machine Learning"[Mesh] OR "Deep Learning"[Mesh] OR "artificial intelligence"[tiab] OR "machine learning"[tiab] OR "deep learning"[tiab] OR "large language model*"[tiab] OR transformer*[tiab] OR "foundation model*"[tiab] OR "AI agent*"[tiab] OR robot*[tiab] OR "natural language process*"[tiab] OR "supervised learning"[tiab] OR "unsupervised learning"[tiab] OR "self-supervised learning"[tiab] OR "reinforcement learning"[tiab]) ) AND ("Eye Diseases"[Mesh] OR "Ophthalmology"[Mesh] OR ocular*[tiab] OR ophthal*[tiab] OR orbital*[tiab] OR intraocular*[tiab] OR "eye disease*"[tiab] OR "eye disorder*"[tiab] OR "eye injur*"[tiab] OR "eye problem*"[tiab] OR fundus[tiab] OR cornea*[tiab] OR conjunctiv*[tiab] OR choroid*[tiab] OR sclera*[tiab] OR retina*[tiab] OR macula*[tiab] OR vitreous[tiab] OR vitreoretinal[tiab] OR "optic disc*"[tiab] OR "optic nerve*"[tiab] OR eyelid*[tiab] OR "extraocular muscle*"[tiab] OR "visual pathway*"[tiab] OR cataract*[tiab] OR glaucoma*[tiab] OR uveitis[tiab] OR iritis[tiab] OR kerato*[tiab] OR keratitis[tiab] OR blepharit*[tiab] OR trachoma*[tiab] OR retinopath*[tiab] OR amblyopia[tiab] OR strabismus[tiab] OR anophthalmia[tiab] OR anisocoria[tiab] OR "optic neuritis"[tiab] OR papilledema[tiab] OR nystagmus[tiab] OR myopia[tiab] OR hyperopia[tiab] OR astigmatism[tiab] OR ametropia[tiab] OR "refractive error*"[tiab] OR "refractive disorder*"[tiab] OR "axial length"[tiab] OR orthokeratology[tiab] OR atropine[tiab] OR blindness[tiab] OR "vision loss"[tiab] OR "visual loss"[tiab] OR "visual impair*"[tiab] OR "vision defect*"[tiab] OR "vision disorder*"[tiab] OR "impaired vision"[tiab] OR "optical coherence tomography"[tiab] OR "fundus photograph*"[tiab] OR "fluorescein angiograph*"[tiab] OR "visual field*"[tiab] OR perimetry[tiab] OR vitrectom*[tiab] OR phacoemulsification[tiab] OR "intravitreal inject*"[tiab] OR "anti-VEGF"[tiab] )) AND (("2015/1/1"[Date - Publication] : "2025/12/31"[Date - Publication]))) AND (English[Language])

2. **Inclusion/exclusion criteria**

Include:

Studies were included if they:

1. Reported AI model, algorithm, or AI-enabled system
2. Applied AI to tasks related to human health or clinical care in the field of ophthalmology

Exclude:

1. Not article/original investigation publication type
2. Animal studies
3. Not AI research

-Exclude studies that use off-the-shelf AI tools for parameter measurement for clinical research

-Exclude studies that mention AI as a future direction

-Exclude studies about rule-based robots or whether its algorithm involves AI is unclear

-Exclude studies that present a dataset for benchmarking and state the potential that the dataset can be used to evaluate AI, but do not do an evaluation

4. Not relevant to ophthalmology

-Exclude pure methodological papers with no clinical data or application, or purely engineering papers about software architecture with no clinical angle

-Exclude studies about AI in biomedical research (e.g. AI for protein structure prediction, machine learning for bioinformatics research)

-Exclude studies about the social or economic impact of AI

-Exclude studies about AI for educational or non-clinical tasks except patient education(e.g. AI for paper writing, AI for medical student education or trainee education)

-Exclude studies that are relevant to many medical specialties or general medicine and ophthalmology is only one of the examples

-Exclude studies that input data for AI models were primarily based on non-ophthalmic imaging (e.g., abdominal CT/MRI for metastasis assessment) or systemic laboratory parameters, rather than ophthalmic imaging or ocular-specific clinical parameters to study topics like liver metastasis of ocular tumor

-Exclude studies that do not pertain to clinical ophthalmology but mention ocular manifestation of other diseases in the paper or the eye as an anatomical organ without involving ocular diseases, clinical examinations, or patient management
