# Supplementary material for: A decade of artificial intelligence research in ophthalmology: Global trends and transferable insights for medical AI
Source: PLOS Digit Health. 2026 Aug 25;5(8):e0001347. doi: 10.1371/journal.pdig.0001347 (PMC13505936; doi:10.1371/journal.pdig.0001347)
Supplement: Supplementary file 2 [file pdig.0001347.s002.docx]

**S2 Table. Labeling Schema for LLM-assisted Classification**

| Classification domain | Permitted label / code | Applicability |
| --- | --- | --- |
| Study scope | specific_disease | Assigned to all included studies. |
|  | anatomical_disease_group* |  |
|  | multiple_general_ophthalmic_diseases* |  |
|  | non_disease_specific |  |
| Specific disease category | diabetic_retinal_diseases | Applied only when study_scope = specific_disease |
|  | macular_and_retinal_degeneration |  |
|  | glaucoma_and_optic_nerve_head_diseases |  |
|  | myopia_and_pathologic_myopia |  |
|  | cataract_and_lens_disorder |  |
|  | corneal_and_ocular_surface_diseases |  |
|  | retinal_vascular_diseases |  |
|  | vitreoretinal_interface_diseases_and_retinal_detachment |  |
|  | inherited_retinal_diseases |  |
|  | neuro_ophthalmic_diseases_and_non_glaucomatous_optic_neuropathies |  |
|  | uveitis_and_intraocular_inflammatory_diseases |  |
|  | ocular_tumor_and_oncology |  |
|  | other_specific_disease |  |
| Study type | model_development_adaptation | Assigned to all included studies. |
|  | existing_locked_model_evaluation |  |
|  | technical_methodological_study |  |
|  | other |  |
| Model paradigm | conventional_machine_learning | Applied only when study_type = model_development_adaptation |
|  | deep_learning |  |
|  | foundation_model |  |
|  | mixed |  |
|  | not_reported |  |
| Input domain | image | Assigned to all included studies. |
|  | text |  |
|  | structured_tabular_clinical_data |  |
| Ophthalmic image modality subtype | image_color_fundus_photography_cfp; image_retinal_oct; image_anterior_segment_oct_as_oct; image_widefield_or_ultra_widefield_fundus_imaging; image_octa; image_slit_lamp_photography; image_visual_field_testing_perimetry_sap; image_corneal_topography; image_corneal_tomography; image_fluorescein_angiography_fa_ffa; image_indocyanine_green_angiography_icga; image_fundus_autofluorescence_faf; image_ultrasound_biomicroscopy_ubm; image_b_scan_ocular_ultrasonography; image_smartphone_based_ophthalmic_imaging; image_other | Applied only when input_image = yes |
| Input modality structure | single_input_unimodality | Assigned to all included studies. |
|  | multi_view_multi_slice_longitudinal_single_modality |  |
|  | within_imaging_multimodality |  |
|  | cross_domain_multimodality |  |

*Note: "anatomical_disease_group" & "multiple_general_ophthalmic_diseases" are combined and coded as "multiple_general_ophthalmic_diseases"
