# Supplementary material for: A decade of artificial intelligence research in ophthalmology: Global trends and transferable insights for medical AI
Source: PLOS Digit Health. 2026 Aug 25;5(8):e0001347. doi: 10.1371/journal.pdig.0001347 (PMC13505936; doi:10.1371/journal.pdig.0001347)
Supplement: Supplementary file 3 [file pdig.0001347.s003.docx]

**S1 Fig. Confusion matrices and Cohen’s κ for human validation of AI-generated annotations across multiple classification tasks**
